# RNA sequencing data of cultured primary rat Müller cells, the spontaneously immortalized rat Müller cell line, SIRMu-1, and the SV40-transformed rat Müller cell line, rMC-1

**DOI:** 10.1016/j.dib.2019.103721

**Published:** 2019-03-07

**Authors:** Thaksaon Kittipassorn, Cameron D. Haydinger, John P.M. Wood, Teresa Mammone, Robert J. Casson, Daniel J. Peet

**Affiliations:** a School of Biological Sciences, Molecular Life Sciences Building, University of Adelaide, Adelaide, SA 5005, Australia; b Department of Ophthalmology and Visual Sciences, Adelaide Health and Medical Sciences Building, University of Adelaide, Adelaide, SA 5000, Australia

## Abstract

Müller cells (MCs), the major type of glial cell of the vertebrate retina, have a vital role in retinal physiology and pathology. They provide structural and functional support for retinal neurons, including photoreceptors, and are implicated in various retinal diseases. Primary and immortalized MCs are important experimental tools for MC research. Here we present high throughput RNA sequencing data of 3 populations of cultured rat MCs: primary cells, the spontaneously immortalized rat MC line, SIRMu-1, and the SV40-transformed rat MC line, rMC-1. These data were deposited in NCBI Gene Expression Omnibus (GEO ID: GSE123161). For data analysis, interpretation and discussion, please refer to the research article, “Characterization of the novel spontaneously immortalized rat Müller cell line SIRMu-1” (Kittipassorn et al., 2019). This dataset is valuable for gaining insight into gene expression profiles of different types of cultured MCs and the roles of MCs in health and disease.

**Table:** Specifications table

Subject area	*Biology, Ophthalmology*
More specific subject area	*Retinal Müller cell (MC) gene expression*
Type of data	*In the article* • *RNA sample information table* *In NCBI Gene Expression Omnibus* • *Raw: fastq files (one per sample)* • *Processed: csv files (one per sample) containing reads per gene (un-normalized and before any filtering)* • *Summary: tsv file containing TMM-normalized* log*2 counts per million reads (cpm) for all samples*
How data was acquired	*mRNA sequencing by an Illumina NextSeq 500 system*
Data format	*Raw and processed data*
Experimental factors	*3 different populations of cultured MCs*
Experimental features	*Total RNA was extracted from cultured primary rat MC cells, the spontaneously immortalized rat MC line, SIRMu-1*[1]*, and the SV40-transformed rat MC line, rMC-1*[2]*. cDNA libraries were prepared from enriched polyA RNA and sequenced.*
Data source location	*Adelaide, Australia*
Data accessibility	*Data available in the article and in NCBI Gene Expression Omnibus*[3]*(GEO ID:*GSE123161*) (*https://www.ncbi.nlm.nih.gov/geo/query/acc.cgi?acc=GSE123161*).*
Related research article	*Kittipassorn, T., Haydinger, C.D., Wood, J.P.M., Mammone, T., Casson, R.J., Peet, D.J., Characterization of the novel spontaneously immortalized rat Müller cell line SIRMu-1, Exp. Eye. Res. 181 (2019) 127-135.**https://doi.org/10.1016/j.exer.2019.01.013*[1]

**Table:** 

**Value of the data** • Commonly and differentially expressed genes can be identified between 3 distinct MC populations, providing invaluable information about the similarity between the different cells, as well as important functional differences. • The rat MC transcriptomic data can be compared with MCs from a range of species from other published analyses to elucidate unique and similar gene expression patterns, signaling pathways and functions of MCs in different organisms. • The ability to determine the origin and nature of a novel cell line. • The data facilitate informed selection based on gene expression of appropriate MC lines to be used as an experimental tool.

## 1 Data

The data presented here are mRNA sequencing analyses of 3 populations of cultured rat MCs: primary cells, the novel spontaneously immortalized rat MC line, SIRMu-1 [1], and the SV40-transformed rat MC line, rMC-1 [2]. RNA samples were extracted from 4 biological replicates of primary MCs, 5 biological replicates of SIRMu-1 cells, and 3 biological replicates of rMC-1 cells. Table 1 in this article provides details on each sample, including the passage number and growth conditions of cells from which the samples were isolated. The data relating to this article are stored in NCBI Gene Expression Omnibus [3] (GEO ID: GSE123161), including: fastq files containing raw sequencing reads (one per sample, 12 in total); csv files containing numbers of reads per gene (un-normalized and unfiltered, one per sample, 12 in total); a tsv file containing TMM-normalized log2 counts per million reads for all 12 samples. Please refer to the related research article [1] for details on data processing.

**Table 1:** RNA sample information.

Cell type	Sample name	Source	Passage no.^a^ of cells used for RNA extraction	Gentamicin and amphotericin B present in culture medium?
Primary MC	P1T1_4	First litter, culture tray 1	4	Yes
	P1T2_4	First litter, culture tray 2	4	Yes
	P2a_3	Second litter	3	Yes
	P3b_3	Third litter	3	Yes
SIRMu-1	S4_6	A frozen vial, passage no. 4	6	No
	S4_11	Same as S4_6	11	No
	S4_11A	Same as S4_6	11	Yes
	S10_11	A frozen vial, passage no. 10	11	No
	S10_20A	Same as S10_11	20	Yes
rMC-1	R22_23	A frozen vial, passage no. 22	23	No
	R22_26	Same as R22_23	26	No
	R24_26	A frozen vial, passage no. 24	26	No

a No., number.

## 2 Experimental design, materials and methods

### 2.1 Cell culture

Primary rat MC cultures were prepared as described previously [1]. Briefly, mixed retinal cultures, consisting of retinal neurons and glial cells, were generated from 2 to 4 day post-natal Sprague-Dawley rat pups. Handling of these animals complied with the Australian Code of Practice for the Care and Use of Animals for Scientific Purposes 2004, and the ARVO Statement for the Use of Animals in Ophthalmic and Vision Research. The cultures were maintained in minimal essential medium (MEM, +Earle's Salts, -L-glutamine, #11090, Life Technologies Australia, Scoresby, VIC, Australia) containing 10% fetal bovine serum (FBS), 87 mg/L gentamicin sulfate, 2.2 mg/L amphotericin B, 25 mM glucose and 2 mM L-glutamine. 25 mM glucose is routinely used in our laboratories with neurons/glial cultures as a lower glucose concentration can lead to problems with cell attachment. After 7 days without any disturbance, the medium of the mixed retinal cultures was changed and the cultures were maintained for 28–42 days with medium changed every 3 days until almost all other cells died, leaving a near homogenous population of MCs. The isolated primary MCs were cultured in the same medium as above but with 20% FBS and passaged at 1:2 when confluent, approximately every 7 days. The SIRMu-1 cell line was cultured in the same medium as the primary MCs with the exception of gentamicin and amphotericin B (unless otherwise stated), and was passaged at 1:4 every 3–4 days. For the rMC-1 cell line (a kind gift of Dr Vijay Sarthy, Northwestern University, Chicago, IL, USA, obtained from Dr Binoy Appukuttan, Flinders University, Adelaide, SA, Australia), the same medium as the SIRMu-1 cells was used but with 10% FBS. rMC-1 cells were passaged at 1:10 every 2–3 days.

### 2.2 RNA extraction

Total RNA was extracted from 4 biological replicates of primary MCs, 5 replicates of SIRMu-1 cells, and 3 replicates of rMC-1 cells (Table 1), using a mirVana miRNA Isolation Kit (#AM1561, Life Technologies Australia) according to the manufacturer's instructions. The antibiotic gentamicin and the antifungal amphotericin B are routinely used to culture primary MCs to prevent contamination as the cells come directly from animals, but these drugs are not used for the culture of immortalized cells. To control for potential differences in transcriptome due to the absence or presence of gentamicin and amphotericin B in culture medium, 2 of the 5 SIRMu-1 samples were extracted from cells grown in medium containing the two drugs, while the other 3 SIRMu-1 samples and all rMC-1 samples were from cells grown without the drugs (Table 1).

### 2.3 Library preparation and mRNA sequencing

RNA samples were submitted to the Australian Cancer Research Foundation (ACRF) Cancer Genomics Facility (Adelaide, SA, Australia). Sample quality was determined on an Agilent 2100 bioanalyzer with an Agilent RNA 6000 Nano kit (#5067-1511, Agilent Technologies, Santa Clara, CA, USA) to confirm that RIN values were above 7 (unless concentrations too low to accurately determine RIN), and concentrations determined using a Qubit RNA HS assay kit (#Q32852, Life Technologies Australia). Libraries were prepared by a KAPA stranded RNAseq HyperPrep kit (#KK8544, KAPA, Cape Town, South Africa) using 5 ng of enriched polyA RNA. RNA was fragmented (approximate insert length: 200 basepairs) and converted to cDNA. End-repair and A-tailing were then performed. Adapters compatible with Illumina sequencing were ligated to the cDNA using a concentration of 1.5 μM with an approximate adapter to molar insert ratio of 200:1. Next, a post-ligation clean-up was carried out to remove excess adapters. The libraries were amplified with 10 cycles of PCR and cleaned with 1X ratio of beads. Library yields and sizes were confirmed on an Agilent 2100 bioanalyzer with an Agilent High Sensitivity DNA kit (#5067-4626, Agilent Technologies) and diluted to 4 nM stocks. Libraries were pooled in equimolar ratios and sequenced using a 75 cycle high output kit (#FC-404-2005, Illumina, San Diego, CA, USA) on an Illumina NextSeq 500 system.
